# The primary care COVID-19 integrated pathway: a rapid response to health and social impacts of COVID-19

**DOI:** 10.1186/s12875-022-01916-3

**Published:** 2022-12-20

**Authors:** Fariba Aghajafari, Brian Hansen, Kerry McBrien, Myles Leslie, Alexandra Chiew, Rick Ward, Bing Li, Jia Hu

**Affiliations:** 1 Department of Family Medicine, Sunridge Family Medicine Teaching Centre, University of Calgary, 2685 - 36 Street NE, Calgary, AB T1Y 5S3 Canada; 2 Primary Care Networks, Calgary, AB Canada; 3 Department of Community Health Sciences, University of Calgary, Calgary, AB Canada; 4 Alberta Health Services, Calgary, AB Canada

**Keywords:** Primary care, Public health, COVID 19, Clinical care pathway

## Abstract

**Background:**

The first wave of COVID-19 in Calgary, Alberta accelerated the integration of primary care with the province’s centrally managed health system. This integration aimed to deliver wraparound in-community patient care through two interventions that combined to create the COVID-19 Integrated Pathway (CIP). The CIP’s interventions were: 1) a data sharing platform that ensured COVID-19 test results were directly available to family physicians (FPs), and 2) a clinical algorithm that supported FPs in delivering in-community follow up to improve patient outcomes. We describe the CIP function and its capacity to facilitate FP follow-up with COVID-19 patients and evaluate its impact on Emergency Department (ED) visits and hospitalization.

**Method:**

We generated descriptive statistics by analyzing data from a Calgary Zone hub clinic called the Calgary COVID-19 Care Clinic (C4), provincially maintained records of hospitalization, ED visits, and physician claims.

**Results:**

Between Apr. 16 and Sep. 27, 2020, 7289 patients were referred by the Calgary Public Health team to the C4 clinic. Of those, 48.6% were female, the median age was 37.4 y. 97% of patients had at least one visit with a healthcare professional, where follow-up was conducted using the CIP’s algorithm. 5.1% of patients visited an ED and 1.9% were hospitalized within 30 days of diagnosis. 75% of patients had a median of 4 visits with their FP.

**Discussion:**

Our data suggest that information exchange between Primary Care (PC) and central systems facilitates primary care-based management of patients with COVID-19 in the community and has potential to reduce acute care visits.

**Supplementary Information:**

The online version contains supplementary material available at 10.1186/s12875-022-01916-3.

## Background

The variable disease course of COVID-19 has resulted in diverse illness, recovery, and healthcare needs among those infected [1–3]. Literature on individuals diagnosed with COVID-19 highlights the need for care pathways in which patients can be monitored by primary care (PC) teams for timely and comprehensive care [1, 4]. Novel pathways incorporating PC have been developed to provide COVID-19 care following hospital discharge [5, 6]. There are studies describing community-based PC pathways for COVID-19 care within a North American context [7–10]. For example, in the United States, a remote monitoring pathway for high-risk COVID-19 patients discharged home was used by new and established PC physicians, medical students, and nurses who conducted daily phone calls to patients and regularly monitored vital signs over eight days, or as needed [5]. Another study used a health information exchange pathway to alert PC teams when veterans were diagnosed with COVID-19 in community facilities [10]. Thus, this model of communication facilitated follow-up care from established PC teams for patients that may not have been identified otherwise [5, 10]. However, there is no literature investigating COVID-19 care models as developed and executed in Canada.

In the present paper we provide an account of a Canadian COVID-19 care pathway, allowing an assessment of the impact Alberta’s COVID-19 Integrated Pathway (CIP) had in the context of Canada’s COVID-19 response and universal healthcare system. The CIP was developed to accelerate PC integration into the broader public health response to the first wave of COVID-19 in the Calgary Health Zone, in Alberta. Specifically, it connected independent family physicians (FPs) with their counterparts in the centrally managed public health system to deliver wraparound patient care. One of the early assumptions to develop the CIP was to manage COVID-19 patients at home, keeping them out of the Emergency Room (ER) and in the Medical Home. Two key elements of the CIP included a data sharing platform that facilitated the provision of COVID-19 test results from provincial public health directly to the relevant FP, and a clinical algorithm [11] that offered PC teams guidance on delivering in-community patient care. ER was aware of the primary care follow up strategy and it changed their threshold for admission and call back: knowing that Primary Care would surveille these patients allowed ERs to discharge 'borderline' patients knowing they would be followed for deterioration. We used clinical and administrative data to evaluate the impact of the pathway on ED visits and hospitalizations, and describe its function and capacity to facilitate FP follow-up with COVID-19 positive patients; and to inform refinement of the CIP for future use.

### Alberta’s primary care context

PC in Alberta is financed directly by the provincial ministry of health with most care delivered by independent family physicians who bill the government on a fee for service (FFS) basis. The fees Alberta’s PC contractors can charge are set in negotiations between the ministry of health and the medical association. Alongside this independent FFS model of PC delivery, the province operates the largest centralized healthcare system in Canada, with over 650 facilities managed by a single health authority: AHS (Alberta Health Services). AHS delivers care in five geographically-based ‘health zones,’ with facilities in these zones providing acute, long term, and some urgent care.

While PC is a highly independent element of the province’s broader system, there are also significant links between it and the central health authority. A PC-focused unit inside AHS is devoted to coordinating, at provincial and zonal levels, the integration of independent PC into the operations of the broader system. With integration of PC and the broader system a provincial policy objective [11], Primary Care Networks (PCNs) serve as key linkage points. The PCNs have evolved into their present form over the last 2 decades [12] and are composed of independent PC physicians who opt in to become members.

The particular mix of programming offered by any given PCN is determined at health zone-level sessions where AHS PC and public health, amongst other central system stakeholders, co-plan to meet the needs of patients and populations in their catchment. Indeed, the PCNs are, legally speaking, joint ventures between the AHS health zones in which they operate and the family physician members who sit on their boards of management. Across Alberta’s health zones the local PCNs have taken different approaches to zonal level planning and service delivery. The Calgary Health Zone contains 7 PCNs, formed by 1700 physician members working in 450 clinics and serving a population of over 1.4 million across urban and rural communities.

## Method

### CIP

**Fig. 1:**
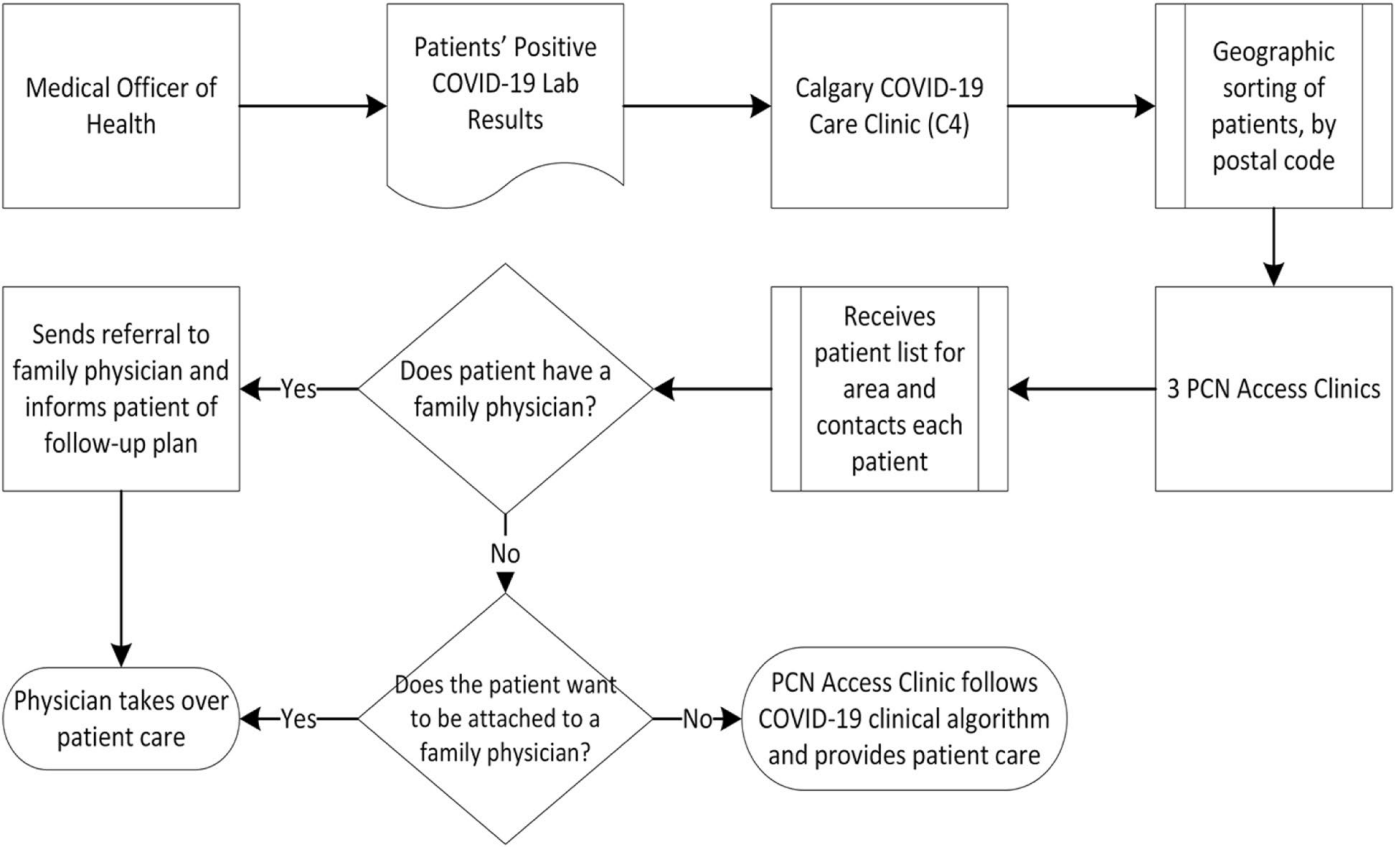
The CIP: a data sharing platform, and a clinical algorithm

The CIP was composed of two interventions: a data sharing platform, and a clinical algorithm (Fig. 1). Early in the pandemic, actions to facilitate the rapid deployment of mass COVID-19 testing in Alberta disrupted established processes whereby FPs would receive notifications about their patients’ lab results. Specifically, the results of tests ordered under the authority of a Medical Officer of Health – as opposed to the patient’s own FP – were not being reported to PC teams. As a result, public health and PC personnel in the Calgary Health Zone created novel integration mechanisms that ensured FPs were alerted of positive COVID-19 cases in their panel and supported in their delivery of treatment and follow-up care. Personnel from Alberta Health Services’ Public Health unit (AHS-PH) and Calgary’s PCNs created a data sharing process that notified PCNs about COVID-19 positive patients as they were referred into the Calgary Health Zone’s hub clinic, called the Calgary COVID-19 Care Clinic (C4). PCNs were then able to contact patients directly and identify if they were attached to an FP and, if so, notify that physician about their patient’s COVID-19 lab result. Patients without an FP were either attached to one or cared for by physicians working in Access Clinics set up by the PCNs (PCN-ACs) directly. This integration of the PCNs and independent FPs into the delivery of care protected the C4 clinic’s capacity to operate and ensured all patients with COVID-19 received follow-up, most of it conducted in-community. Personnel from the PCNs and AHS also developed the Calgary Zone COVID-19 clinical algorithm based on the current evidence available on COVID-19 [13]. This clinical algorithm supported FPs to provide both medical and social care to COVID-19 positive patients in the community. Using the algorithm’s risk stratification tool, family physicians identified patients at low, medium, and high risk of complications, and using the treatment algorithm mapped out a care plan. The algorithm also provided family physicians with linkages to public health, specialists, and acute care [14]. The algorithm was regularly updated based on the emergence of new evidence. The PCN leadership provided multiple informational webinars for PCN physicians on the use of CIP.

### Data source, setting, and study population

We obtained data on patients’ COVID-19 status and characteristics from the C4 clinic. Every patient in the Calgary Health Zone who was suspected of having COVID-19 was referred to the C4 clinic in the first and second waves of the pandemic. We used the data from the first wave, between April 16-September 27, 2020. Additional data were drawn from records of the Provincial Lab, Alberta Health Registry, Discharge Abstract Database (DAD), National Ambulatory Care Classification Reporting System (NACRS), and Practitioner Claims. A unique patient identifier (provincial healthcare number – PHN) was used to link patients’ COVID-19 status to the administrative data sources to capture clinical information, and encounters with acute and primary healthcare services. These data were used to determine the rate of hospitalization, ED visits, and number of FP visits for each patient.

### Statistical analysis

We summarized patient demographic and clinical characteristics using descriptive statistics, means and standard deviations (SD), median and interquartile range 25^th^-75^th^, and proportions for the overall cohort. We also determined the number of hospital admissions and ED encounters as well as FP visits for the overall cohort. SAS was used for all analyses (SAS 9.4) [15]. This project was approved by the University of Calgary Conjoint Health Research Ethics Board (REB20-0959_MOD5).

## Results

### Baseline demographics

Between April 16 and September 27, 2020, 7706 cases who were confirmed/ suspicious for COVID-19 were referred to the C4 clinic by AHS Public Health. Sixty seven patients were removed from the dataset due to missing PHNs. The remaining cases were linked to Alberta Health Care Insurance plan (AHCIP) data to validate the patients’ date of birth, residential postal code, and sex, leaving 7289 patients with valid data, 350 patients did not have validated data. C4 clinic staff were not able to contact 182 patients, but the rest (97.5%) had at least one phone call with a C4 clinic physician or a nurse. The mean age of the patients was 37.4 (19.6) (range: 0.1–105); 59% were between 40 and 60, and 28% were over 60 years of age. There were 3539 (48.6%) female patients (Table 1).

**Table 1:** Characteristics of patients

Total number of patients	N = 7289
Mean age ± SD	37.4 (19.6)
Age range, y	(0.1–105.6)
Age < 40	4300 (59%)
40–60	2041 (28%)
≥ 60	948 (13%)
Female	3539 (48.6%)
Male	3750 (51.4%)
Hospitalization	141 (1.9%)
ED visit- 30 days	370 (5.1%)
GP visit- 30 days	5533 (76%)
Average number of GP visit	(21,940/5533 = 3.97)
Average number of GP visit < 40	3.23
Average number of GP visit 40–60	4.33
Average number of GP visit ≥ 60	6.01

#### Patients’ flow and follow up

**Fig. 2:**
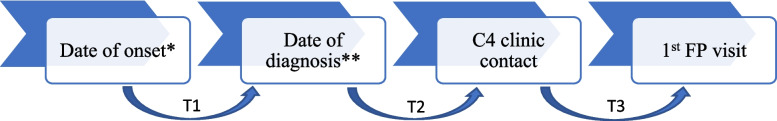
Patient’s flow

Figure 2 shows the flow of patient information used to facilitate follow-up care from April 16 to September 27, 2020, in Calgary. The median (25^th^-75th) time from the date of onset to the date of diagnosis (T1) was 3 (1–6) days, from the date of diagnosis to C4 clinic contact (T2) was 1 (1–2) day and from the C4 clinic contact to first FP visit was 1 (0–4) day.

**Fig. 3:**
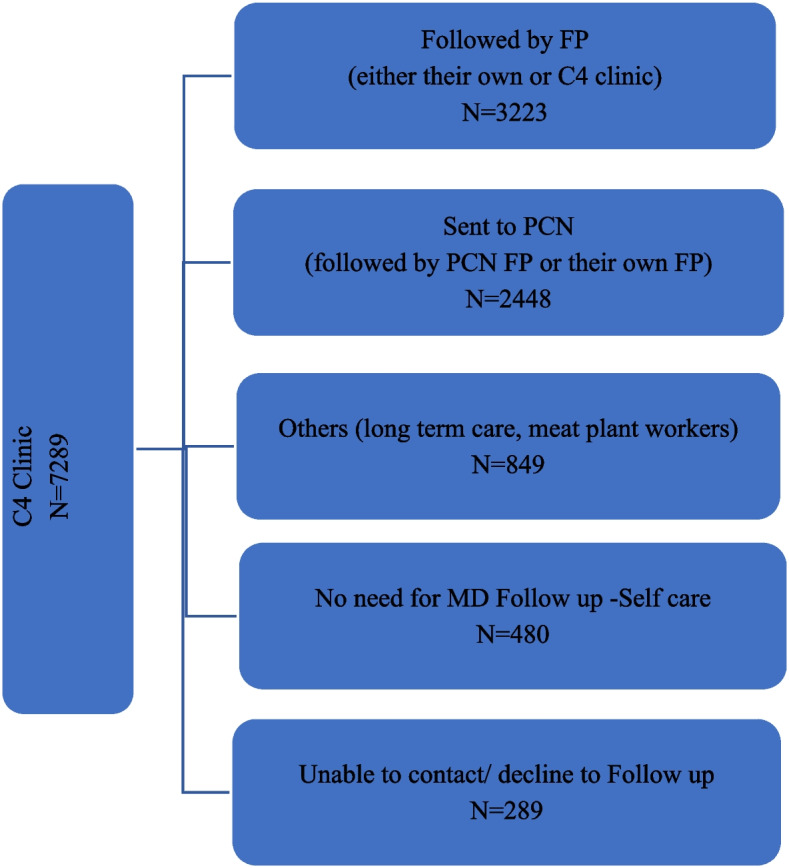
Patient distribution form C4 clinic

Figure 3 shows the follow up distribution of patients from C4 clinic. Overall, 44.2% of patients followed by either their own FP (upon availability of patient’s FP) or the C4 Clinic FP, 33.6% of patients were referred to PCN Access clinics and were followed either by their own FP from there (upon availability of patient’s FP) or Access clinic FP, and 11.6% patients were from Long Term Care (LTC) facilities or foreign workers from meat plant outbreak.

There were 21,940 FP visits logged within 30 days after data were received by the C4 Clinic among 5533 patients (76% of patients had a visit with FP). Patients had an overall average of 3.97 FP visits. The average number of visits was 3.23 for patients younger than 40 years, 4.33 for patients between 40–60 years and 6 for patients over 60 years of age.

#### ED visit and hospitalization

Overall, there were 463 ED visits among 370 COVID-19 positive patients within 30 days after C4 Clinic contact (5.1%). 80% of ED visits were due to shortness of breath, chest pain, cough, or abdominal pain.

There were 150 hospitalizations from 141 patients among 7289 COVID-19 positive patients within 30 days after C4 Clinic contact (1.9%). There were 15 deaths among hospitalized patients. 75% of hospitalizations were due to confirmed COVID-19 (virus identified).

## Discussion

The CIP consists of two interrelated interventions: 1) A data sharing platform that disseminated COVID-9 lab test results from the province’s public health system to local PC providers, and 2) A clinical algorithm that offers those providers guidance for patient care. In combination, the CIP’s interventions introduced a data integrated and standardized approach to care planning and delivery across the Calgary Health Zone. These system changes enabled a more coordinated and efficient response to the pandemic and ensured all patients received PC follow-up. We showed that during wave one of the pandemic, almost every patient was contacted through the AHS-run C4 hub clinic and, based on their risk, received timely follow up care – as structured by the CIP – either with a PCN-access clinic physician or their own FP. The median time from positive COVID-19 test result reporting to follow-up from the C4 clinic was 1 day, and from follow-up from the C4 clinic to a FP visit was a further 1 day.

The purpose of the CIP is to facilitate care of COVID-19 positive patients by PC teams in the community and to decrease the use of acute care. We showed that the rate of ED visits and hospitalizations within 30 days after C4 Clinic contact was 5.1% and 1.9%, respectively. We do not have a control group to compare these numbers to; however, Canadian data from the Public Health Agency of Canada (PHAC) and the Canadian Institute for Health Information on ED visits and hospitalization rates from April – September 2020 was 30.5% and 10.3%, respectively (Appendix A) [16, 17]. This could indicate that use of the CIP in the Calgary Zone may have decreased ED visits and hospitalization during wave one. However, we acknowledge that most new COVID-19 cases reported between April and September 2020 were in the provinces of Quebec and Ontario (79.81%) with only 11.25% of cases from Alberta [18], which may be due to a lower proportion of older adults with COVID-19 in Alberta [18, 19]. Provincial-level data and data from PHAC showed that as compared to Ontario, Alberta contained a lower proportion of COVID positive patients aged 50 years and older (43% vs 25%) between April-September 2020 [18, 19], as well as a lower number of hospitalized patients in Intensive Care Unit (ICU) compared to Ontario and Quebec (0.14% vs. 1.07% vs.1.53%) [20] (Appendix A). In addition, the rate of ED visits and hospitalization in this data set is from patients in the community and did not include patients from LTC where most hospitalization and death occurred.

Literature evaluating community-based COVID-19 care pathways in the United States demonstrates similar findings [5, 10]. Patel et al. evaluated a remote monitoring pathway for high-risk COVID-19 patients discharged home in Colorado [5]. This pathway was delivered by new and established PC physicians, medical students, and nurses who conducted daily phone calls to patients and regularly monitored vital signs over eight days, or as needed [5]. Between April and June 2020, 422 patients were monitored through the pathway, with only 4% re-admitted to the hospital and 3% visiting the emergency department within 30 days [5].

Sherman et al. described a health information exchange pathway in the United States which alerts PC teams when veterans are diagnosed with COVID-19 in community facilities [10]. The health information exchange system integrated COVID-19 results from state reporting systems to identify and note COVID-19 diagnoses on patients’ electronic medical records, notifying their PC team for follow-up [10]. Over a three-month period, the information exchange pathway effectively facilitated clinician follow-up for 76% of Veterans diagnosed with COVID-19 in the community, with 63% receiving follow-up from their established primary care team [10]. The importance of information exchange between PC and central systems, emphasized by Sherman et al [10], is confirmed by our analysis.

In addition, Ganesh et al. reported that among 849 COVID-19 positive patients engaged with a virtual care model, only 8.9% had an ED visit within 60 days, based on recommendations from care team physicians due to severe COVID-19 symptoms [9]. Of those, 40% were subsequently hospitalized, with 36% requiring admission to an intensive care unit [9]. Kerkhoff et al. similarly reported that a community-based care model for vulnerable COVID-positive Latinx patients resulted in 3 of 80 patients being directed to the ED due to severe COVID-19 symptoms, with only 1 hospitalized [7]. However, only 10% of patients engaged with this care model were connected to primary care within one month, which may be due to population and contextual differences [7]. The low levels of acute care use suggest such pathways may be useful in diverting patients from acute care settings. This is consistent with a study by Ye et al., which identified a trend towards reduced 14-day ED visits among COVID-19 patients referred to a remote monitoring pathway post-discharge, compared to those without remote monitoring [6]. These findings are aligned with our study that showed using CIP potentially decreased acute care visits in patients with COVID-19.

The CIP is an intervention developed in the Calgary Health Zone to facilitate the flow of data about, and provision of primary care to, patients with COVID-19, and it represents a model of collaboration between primary care, specialty care, and public health. Collaborative partnerships between distinct sectors allow for more effective action on an issue than if each sector were acting alone [21]. Specifically, collaborations between primary care and public health sectors have gained interest over the years, with the pandemic emphasizing their importance in Canadian contexts [22, 23].

Within Canada, primary care is often the first point of entry to the healthcare system, providing tailored and patient-centered care, whereas public health supports societal conditions that promote better health [24]. Though during the pandemic, public health has played a crucial role in COVID-19 identification, contact tracing, and information sharing [25], and can thus facilitate primary care follow-up for illness management within the community [26]. Together, primary care and public health collaborations provide an integrated approach to improving both individual and population health [24]. Literature reports that successful collaborations are beneficial for community members and those involved in the collaboration [24].

Our study has several limitations that should be considered. Additional data collected from PCNs to measure the time from a patient’s follow-up with the C4 clinic to follow-up by a PCN physician were incomplete and unable to be used. Secondly, this study did not have a control group to be able to definitively conclude that the CIP reduced ED visits and hospitalizations. Thirdly, there may be some possible negative impacts of the pathway, for example, some patients felt that they received too many calls from the nurses and physicians, however, the negative impact of the pathway could not be measured with the existing data sources. However, our qualitative enquiry (presented somewhere else) showed that physicians and nurses were satisfied with using CIP for the following reasons: providing clear and rapid information in a rapidly changing context, providing clinical guidance on how to best support patients managing their symptoms at home, connecting patients to Specialists as required, managing symptom trajectory, connecting patients to other resources (basic needs, psychosocial, etc.) as required. At the same time, CIP allowed for enough physician clinical judgement along with standardized intervention. Finally, we were not able to measure a number of social determinants of health that are not captured within our administrative data sources and could affect the continuity of care, ED visit and hospitalization.

## Conclusion

Our study is a quantitative approach evaluating an integrated care model (CIP) to provide care during pandemic. Our data suggest that that information exchange between PC and central systems facilitates primary care-based management of patients with COVID-19 in the community and has potential to reduce acute care visit. The CIP continues to be used during subsequent waves of COVID-19 and is now part of a broader, province-wide care pathway in Alberta.

## Supplementary Information

**Additional file 1: **Appendix A. COVID-19 Acute Care Use **Supplementary Table 1.** Proportion of COVID-19 hospitalizations in Canada by month, April-September 2020, based on total COVID-19 cases. **Supplementary Table 2.** Proportion of COVID-19 emergency department visits in Canada by month, April-September 2020, based on total COVID-19 cases. **Supplementary Table 3.** COVID-19 cases in Ontario between April-September 2020, by age group. **Supplementary Table 4.** COVID-19 cases in Alberta between April-September 2020, by age group. **Supplementary Table 5.** COVID-19 case severity between April-September 2020, by age group.

## Data Availability

The datasets analysed during the current study are not publicly available due to being the property of Alberta Health Services, but are available from the corresponding author on reasonable request.

## Abbreviations

CIP: COVID-19 Integrated Pathway
FPs: Family Physicians
C4: Calgary COVID-19 Care Clinic
ED: Emergency Department
PC: Primary Care
ER: Emergency Room
AHS-PH: Alberta Health Services’ Public Health unit
PCNs: Primary Care Networks
DAD: Discharge Abstract Database
NACRS: National Ambulatory Care Classification Reporting System
PHN: Provincial Healthcare Number
SD: Standard Deviations
AHCIP: Alberta Health Care Insurance plan
LTC: Long Term Care
PHAC: Public Health Agency of Canada
