# Supplementary material for: The primary care COVID-19 integrated pathway: a rapid response to health and social impacts of COVID-19
Source: BMC Prim Care. 2022 Dec 20;23:333. doi: 10.1186/s12875-022-01916-3 (PMC9765374; doi:10.1186/s12875-022-01916-3)
Supplement: Supplementary file 1 — Additional file 1: Appendix A. COVID-19 Acute Care Use Supplementary Table 1. Proportion of COVID-19 hospitalizations in Canada by month, April-September 2020, based on total COVID-19 cases. Supplementary Table 2. Proportion of COVID-19 emergency department visits in Canada by month, April-September 2020, based on total COVID-19 cases. Supplementary Table 3. COVID-19 cases in Ontario between April-September 2020, by age group. Supplementary Table 4. COVID-19 cases in Alberta between April-September 2020, by age group. Supplementary Table 5. COVID-19 case severity between April-September 2020, by age group. [file 12875_2022_1916_MOESM1_ESM.docx]

**Appendix A. COVID-19 Acute Care Use**

Supplementary Table 1. Proportion of COVID-19 hospitalizations in Canada by month, April-September 2020, based on total COVID-19 cases

| **Month** | **Apr 2020** | **May 2020** | **Jun 2020** | **Jul 2020** | **Aug 2020** | **Sep 2020** | **Total**  **(Apr-Sep 2020)** |
| --- | --- | --- | --- | --- | --- | --- | --- |
| **Hospitalizations (#)** | 2,650 | 2,101 | 1,317 | 893 | 585 | 696 | 8,242 |
| **Total New COVID-19 Cases (#)** | 21,312 | 14,190 | 8,858 | 8,254 | 9,456 | 18,014 | 80,084 |
| **Percent Hospitalized (%)** | 12.43 | 14.81 | 14.87 | 10.82 | 6.19 | 3.86 | 10.29 |

*Note: Total COVID-19 cases = confirmed + probable COVID-19 cases; Hospitalization data excludes Quebec; New cases = newly identified COVID-19 cases, does not include cumulative numbers up to date. Data on COVID-19 cases obtained from the Public Health Agency of Canada^13^ and data on COVID-19 hospitalizations obtained from the Canadian Institute for Health Information^14^.*

Supplementary Table 2. Proportion of COVID-19 emergency department visits in Canada by month, April-September 2020, based on total COVID-19 cases

| **Month** | **Apr 2020** | **May 2020** | **Jun 2020** | **Jul 2020** | **Aug 2020** | **Sep 2020** | **Total**  **(Apr-Sep 2020)** |
| --- | --- | --- | --- | --- | --- | --- | --- |
| **ED Visits (#)** | 13,727 | 10,235 | 5,085 | 4,418 | 3,747 | 8,620 | 45,832 |
| **Total New COVID-19 Cases (#)** | 44,688 | 37,711 | 13,257 | 12,108 | 12,636 | 29,810 | 150,210 |
| **Percent Visited ED (%)** | 30.72 | 27.14 | 38.36 | 36.49 | 29.65 | 28.92 | 30.51 |

*Note: ED = Emergency department; Total COVID-19 cases = confirmed + probable COVID-19 cases; Quebec data is included for ED visits only; New cases = newly identified COVID-19 cases, does not include cumulative numbers up to date. Data on COVID-19 cases obtained from the Public Health Agency of Canada^13^ and data on COVID-19 emergency department visits obtained from the Canadian Institute for Health Information^14^.*

**COVID-19 Cases by Age Group in Ontario in Alberta**

Supplementary Table 3. COVID-19 cases in Ontario between April-September 2020, by age group

| **Age Group** | **Proportion of COVID Cases – no. (%)** | **% Fatal** |
| --- | --- | --- |
| <20 years | 4329 (8.7%) | 0.02% |
| 20s | 9939 (20.1%) | 0.04% |
| 30s | 7416 (15.0%) | 0.11% |
| 40s | 6706 (13.5%) | 0.33% |
| 50s | 7224 (14.6%) | 1.19% |
| 60s | 4825 (9.7%) | 5.33% |
| 70s | 2975 (6.0%) | 16.54% |
| 80s | 3618 (7.3%) | 28.91% |
| 90+ years | 2525 (5.1%) | 37.50% |
| Unknown | 3 (0.01%) | 0.00% |
| All | 49,560 (100%) | 5.78% |

*Note:* Data obtained from the Government of Ontario^18^

Supplementary Table 4. COVID-19 cases in Alberta between April-September 2020, by age group

| **Age Group** | **Proportion of COVID Cases – no. (%)** | **% Fatal** |
| --- | --- | --- |
| Under 1 year | 121 (0.7%) | 0.00% |
| 1-4 years | 499 (2.9%) | 0.00% |
| 5-9 years | 662 (3.8%) | 0.00% |
| 10-19 years | 1840 (10.6%) | 0.00% |
| 20-29 years | 3233 (18.7%) | 0.00% |
| 30-39 years | 3417 (19.8%) | 0.03% |
| 40-49 years | 3021 (17.5%) | 0.03% |
| 50-59 years | 1996 (11.5%) | 0.30% |
| 60-69 years | 1251 (7.2%) | 1.60% |
| 70-79 years | 613 (3.5%) | 9.79% |
| 80+ years | 647 (3.7%) | 25.04% |
| Unknown | 1 (0.01%) | 0.00% |
| All | 17,301 (100%) | 1.45% |

*Note:* Data obtained from the Government of Alberta^19^

**COVID-19 Cases by hospitalization and death Group in Canada**

Supplementary Table 5. COVID-19 case severity between April-September 2020, by age group

|  | **Quebec**  **(n=19,060)** | **Ontario and Nunavut (n=14,251)** | **Prairies (Alberta, Saskatchewan, and Manitoba) and the Northwest Territories (n=5,798)** |
| --- | --- | --- | --- |
| **Asymptomatic – no. (%)** |  |  |  |
| Yes | 3,097 (16.25%) | 2832 (19.87%) | 958 (16.52%) |
| No | 15,933 (83.59%) | 10,810 (75.85%) | 4,332 (74.72%) |
| Not stated | 30 (0.16%) | 609 (4.27%) | 508 (8.76%) |
| **Hospital Status – no. (%)** |  |  |  |
| Hospitalized and in intensive care unit | 292 (1.53%) | 153 (1.07%) | 8 (0.14%) |
| Hospitalized, but not in intensive care unit | 1532 (8.04%) | 888 (6.23%) | 202 (3.48%) |
| Not hospitalized | 17,229 (90.39%) | 4,535 (31.82%) | 5,563 (95.95%) |
| Not stated/Unknown | 7 (0.04%) | 8,675 (60.87%) | 25 (0.43%) |
| **Death – no. (%)** |  |  |  |
| Yes | 1457 (7.64%) | 657 (4.61%) | 43 (0.74%) |
| No | 17603 (92.36%) | 13,594 (95.39%) | 5,753 (99.22%) |
| Not stated | - | - | 2 (0.03%) |

*Note:* Data obtained from the Public Health Agency of Canada^20^
